# Growth Response of Drought-Stressed *Pinus sylvestris* Seedlings to Single- and Multi-Species Inoculation with Ectomycorrhizal Fungi

**DOI:** 10.1371/journal.pone.0035275

**Published:** 2012-04-05

**Authors:** Tabea Kipfer, Thomas Wohlgemuth, Marcel G. A. van der Heijden, Jaboury Ghazoul, Simon Egli

**Affiliations:** 1 Forest Dynamics, Swiss Federal Research Institute WSL, Birmensdorf, Switzerland; 2 Ecosystem Management, Institute of Terrestrial Ecosystems, ETH Zürich, Zürich, Switzerland; 3 Ecological Farming Systems, Agroscope Reckenholz-Tänikon, Zürich, Switzerland; 4 Institute of Evolutionary Biology and Environmental Studies, University of Zürich, Zürich, Switzerland; 5 Plant–Microbe Interactions, Institute of Environmental Biology, Faculty of Science, Utrecht University, Utrecht, The Netherlands; USDA-ARS, United States of America

## Abstract

Many trees species form symbiotic associations with ectomycorrhizal (ECM) fungi, which improve nutrient and water acquisition of their host. Until now it is unclear whether the species richness of ECM fungi is beneficial for tree seedling performance, be it during moist conditions or drought. We performed a pot experiment using *Pinus sylvestris* seedlings inoculated with four selected ECM fungi (*Cenococcum geophilum*, *Paxillus involutus*, *Rhizopogon roseolus* and *Suillus granulatus*) to investigate (i) whether these four ECM fungi, in monoculture or in species mixtures, affect growth of *P. sylvestris* seedlings, and (ii) whether this effect can be attributed to species number *per se* or to species identity. Two different watering regimes (moist *vs.* dry) were applied to examine the context-dependency of the results. Additionally, we assessed the activity of eight extracellular enzymes in the root tips. Shoot growth was enhanced in the presence of *S. granulatus*, but not by any other ECM fungal species. The positive effect of *S. granulatus* on shoot growth was more pronounced under moist (threefold increase) than under dry conditions (twofold increase), indicating that the investigated ECM fungi did not provide additional support during drought stress. The activity of secreted extracellular enzymes was higher in *S. granulatus* than in any other species. In conclusion, our findings suggest that ECM fungal species composition may affect seedling performance in terms of aboveground biomass.

## Introduction

Tree roots are usually colonized by a wide range of microbes, including those fungi that form ectomycorrhizas (ECM). Ectomycorrhizal fungi provide nutrients and water to trees in exchange for carbohydrates [1]. Global change, however, is expected to alter ECM fungal communities. Shifts in community composition and decreases in species number have been found as a consequence of anthropogenic nitrogen deposition [2], elevated CO_2_ [3], [4], drought [5] and disturbances such as forest fire [6]. For example, after forest fires in the dry inner-alpine valleys of the Central Alps, a reduction in ECM fungal species number and changes in fungal species composition were found [7]. Simultaneously, regeneration failure of *Pinus sylvestris* after fire disturbance has been reported, and seedling mortality has been attributed to drought [8]. It is hypothesized that ECM fungi may play a crucial role for *P. sylvestris* tree regeneration, especially under adverse climatic conditions, given that this tree species obligately depends on fungal symbionts [9], [10]. The consequences of a reduction in ECM fungal species richness and changes in ECM community composition for tree performance, however, are poorly explored.

The theoretical framework to explain the mechanisms of a positive relationship between plant species richness and plant productivity has been developed based on experiments with plants from grassland ecosystems [11], [12]. Two major concepts emerged: species complementarity and sampling or selection probability effect. Basically, complementarity refers to differences in resource requirements between plant species such that community production is raised through a more efficient use of total available resources. A sampling or selection probability effect denotes the fact that species rich plant communities are more likely to have a higher yield on account of an increased probability that a species with a high growth rate at given environmental conditions is included in that community [13]. This is indicated when an individual plant species generates similar or greater responses in plant productivity than species mixtures. In contrast, effects of diversity *per se* are suggested when the productivity of species mixtures outperforms that of any species monoculture (overyielding [14]). We hypothesize that these concepts may also hold for the relationship of ECM fungal richness and host productivity. The association of a plant with fungal species of different morphological and physiological attributes may provide access to a broader range of nutrient pools [1], [15], and a larger number of species of ECM fungi may also increase the probability of a fungal isolate being present that particularly promotes plant growth. Evidence for a positive species richness-productivity relationship exists for arbuscular mycorrhizal fungi (AMF). Shoot biomass or total biomass of the host has been shown to increase with increasing number of AM species, and this has been explained by a sampling effect [16], [17], a complementarity effect due to AMF species of different phylogenetic groups that were included [18] or a combination of both effects [19]. To our knowledge, only two studies investigated the species richness-productivity relationship in ECM, and their results are inconsistent. In the experiment by Baxter & Dighton [20], inoculation of *Betula populifolia* in Petri dishes resulted in an increase in root biomass with increasing species richness, but in a decrease in shoot biomass. In the pot experiment of Jonsson et al. [21], the outcome of the ECM species richness treatments depended on soil fertility (high *vs.* low), host species (*Betula pendula* *vs.* *P. sylvestris*) and plant parts (shoots *vs.* roots).

Functional variation between taxa of ECM fungi is documented for nutrient source utilisation in terms of nitrogen [22], [23] and phosphorus [24], which is a prerequisite for niche complementarity. Many relevant enzymatic capabilities differ between ECM species, and profiling of eight key extracellular enzymes involved in degradation of celluloses, hemicelluloses, chitin and proteins, the oxidation of phenols and the mobilization of phosphorus has proved to be a useful tool to characterize fungal species in terms of their capabilities for nutrient acquisition under given environmental conditions [25], [26].

Besides improving plant nutrition ECM fungi also enhance water uptake under dry conditions. The fungal mycelium can explore a larger volume of soil than non-mycorrhizal roots, and hyphae can enter small soil pores that are not accessible to the short roots of a plant [27], [28] thus improving plant water relations under low water conditions [29], [30], [31]. Some studies, however, have found no beneficial effect of ECM, suggesting that both symbionts are directly limited by water availability [32], [33]. Fungal growth might be reduced by drought and consequently, the ability of the fungus to supply the plant with nutrients declines. Simultaneously, the plant responds to drought, and C flow to the roots and the fungal symbionts ceases. It has been experimentally shown that plants’ response to drought varies considerably depending on the associated fungal species [32], [34]. Therefore, we hypothesize that water availability affects the outcome of the ECM symbiosis, and especially whether a positive relationship between fungal species richness and host productivity occurs. To test this hypothesis, we potted *Pinus sylvestris* seedlings and four selected ECM fungi (*Cenococcum geophilum*, *Paxillus involutus*, *Rhizopogon roseolus* and *Suillus granulatus*) in all possible species combinations and grew them under two moisture conditions. Shoot and root biomass of *P. sylvestris* seedlings was assessed as well as the activity of eight key extracellular enzymes in excised root tips colonized with one of the fungal species. The following questions were addressed: (i) How do ECM fungi, in monoculture or in species mixtures, affect *P. sylvestris* seedling growth, and can this be attributed to species number *per se* or does it depend on species identity? (ii) If there is an effect of ECM fungi on seedling biomass, can it be attributed to the activity of extracellular enzymes of the root tips? (iii) Do the effects of ECM fungi on seedling biomass differ under dry and moist conditions, respectively?

## Results

**Figure 1:**
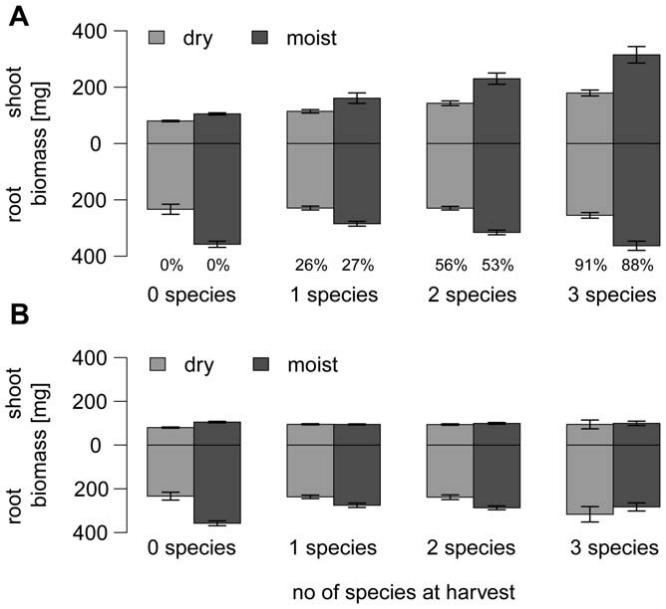
Biomass (mean ± standard error) of *Pinus sylvestris* seedlings with different combinations of ECM fungal species. Panel A shows all fungal species combinations and panel B combinations where *S. granulatus* was absent. Abbreviations: 0 species = non-mycorrhizal samples; 1 species = monocultures with *Cenococcum geophilum*, *Paxillus involutus*, *Rhizopogon roseolus* and *Suillus granulatus*, respectively; 2 and 3 species = multiple combinations of the four species containing two or three species, respectively. The species numbers refer to the status at harvest, as not all fungal species initially inoculated actually associated with seedling roots. The numbers below the bars in panel A indicate the percentage of samples containing *S. granulatus*.

**Figure 2:**
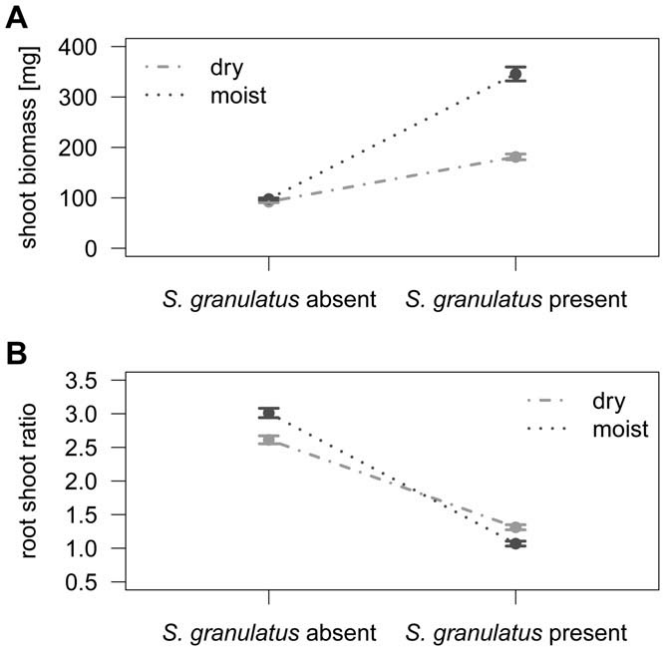
Interaction plot for shoot biomass (A) and root/shoot ratio (B) of *Pinus sylvestris* seedlings. Seedlings were grown with or without *Suillus granulatus* and under two watering regimes (dry and moist, respectively). Presence/absence of *S. granulatus* refer to the status at harvest. Whiskers indicate standard errors.

**Figure 3:**
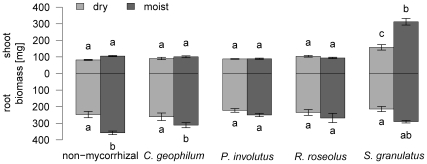
Biomass of *Pinus sylvestris* seedlings grown with monocultures of ECM fungi or without fungi. Bars display mean values and whiskers depict the corresponding standard errors. Different letters indicate significant differences between treatments (*P* < 0.05, Tukey’s HSD test).

ECM fungi enhanced shoot growth of *Pinus sylvestris* seedlings under both moist and dry conditions (Figure 1A; GLM on presence of ECM, estimate = 0.537, *P* = 0.006). The increased aboveground productivity of ECM seedlings was caused by one species, *Suillus granulatus* (Table 1). *Cenococcum geophilum*, *Paxillus involutus* and *Rhizopogon roseolus* did not affect shoot biomass, neither under dry nor moist conditions (Figure 1B; GLM on presence of ECM, subset of data without *S. granulatus*, estimate = 0.092, *P* = 0.116). The effect of *S. granulatus* was more pronounced in the moist treatment than in the dry treatment (significant interaction term in GLM, Table 1 and Figure 2A). Under moist conditions, the presence of *S. granulatus* caused an increase in shoot biomass from 105 ± 9 mg (non-mycorrhizal seedlings) to 312 ± 7 mg (monocultures of *S. granulatus*), whereas under dry conditions, shoot biomass increased from 82 ± 11 mg to 157 ± 6 mg (mean±standard error; Figure 3). The root/shoot ratio decreased from 3±0.07 (*S. granulatus* present) to 1.1 ± 0.04 (*S. granulatus* present) under moist conditions, but merely dropped from 2.6 ± 0.06 to 1.3 ± 0.04 under dry conditions (mean±standard error; significant interaction term in GLM, estimate = 0.356, *P* < 0.01; Figure 2B). The positive relationship between species number and shoot biomass depicted in Figure 1A can be explained by the fact that with increasing species number, a higher proportion of samples are containing the growth-promoting species *S. granulatus* (numbers written below the bars indicate the percentage of samples containing *S. granulatus*). This sampling effect is corroborated by the fact that there was no overyielding: there was no significant difference in shoot biomass between seedlings grown in 3-species mixtures compared to that of seedlings grown in monocultures of *S. granulatus*, the fungal species with the highest yielding (two-sided *t*-tests with Holm’s correction, *P* > 0.05).

**Table 1:** GLM summary for the relationship between shoot biomass of *Pinus sylvestris* seedlings (response) and species identity, watering regime and their interaction.

Effect	Estimate	p-value
Watering regime (dry condition)	–0.012	0.488
Absence of ectomycorrhiza	0.022	0.307
Presence of *Cenococcum geophilum*	–0.001	0.908
Presence of *Paxillus involutus*	0.014	0.136
Presence of *Rhizopogon roseolus*	–0.006	0.464
Presence of *Suillus granulatus*	0.242	**<0.001** *
Watering x absence of ectomycorrhiza	–0.049	0.097
Watering x *Cenococcum geophilum*	0.005	0.684
Watering x *Paxillus involutus*	–0.001	0.945
Watering x *Rhizopogon roseolus*	0.010	0.438
Watering x *Suillus granulatus*	–0.109	**<0.001** *

* Significant p-values (<0.01) are given in bold.

D^2^ = 0.841.

**Figure 4:**
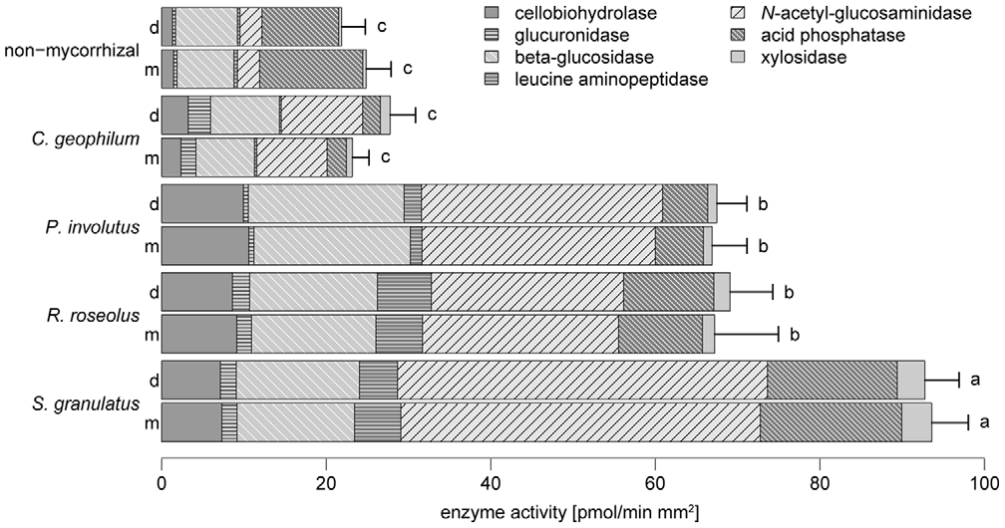
Enzyme activity in root tips from *Pinus sylvestris* seedlings. Seedlings were non-mycorrhizal or infected with monocultures of *Cenococcum geophilum*, *Paxillus involutus*, *Rhizopogon roseolus* or *Suillus granulatus*, respectively, and grown under two watering regimes (d = dry, m = moist). Different letters indicate significant differences in overall enzyme activity between treatment combinations (*P*<0.05, Tukey’s HSD test). Whiskers indicate standard errors of overall enzyme activity.

Overall enzyme activity was higher in *S. granulatus* root tips than in *C. geophilum* or non-mycorrhizal root tips and intermediate in *R. roseolus* and *P. involutus* (Figure 4). There was no difference in enzyme activity between the two watering regimes, but the enzyme profile of the mycorrhizal roots differed from the non-mycorrhizal roots. In mycorrhizal root tips, the activity of *N*-acetyl-glucosaminidase (a chitinase) was high, especially in *S. granulatus*, whereas acid phosphatase activity was comparatively high in non-mycorrhizal root tips.

**Figure 5:**
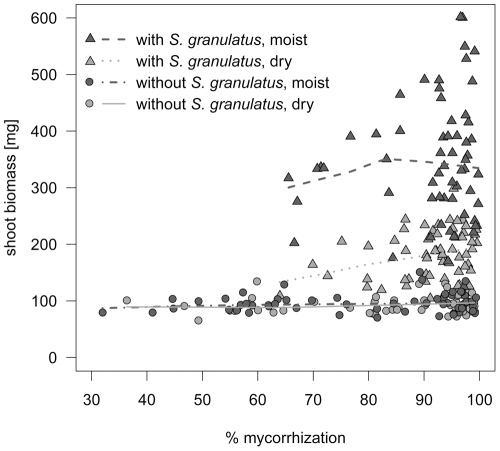
Relationship between % mycorrhization and shoot biomass in *Pinus sylvestris* seedlings. Seedlings were grown in the presence and absence of *Suillus granulatus*, respectively, and under dry and moist conditions. Presence/absence of *S. granulatus* refer to the status at harvest. Lines were drawn based on LOWESS smoothing.

Mycorrhization of the roots was generally high: 75% of the samples had colonization rates between 80 and 100%, and there were no differences between moist and dry treatments (two-sided Welch *t*-test, *P* = 0.892). Samples with low colonization rates were mostly monocultures with *C. geophilum* (data not shown), whereas cultures containing *S. granulatus* had colonization rates above 64%. Shoot biomass was independent of mycorrhizal colonization if the effects of *S. granulatus* and soil moisture were taken into account (Figure 5).

## Discussion

We found that the presence of one specific fungus, *Suillus granulatus*, considerably increased aboveground biomass of *Pinus sylvestris* seedlings compared to non-mycorrhizal plants. In contrast, *Cenococcum geophilum*, *Paxillus involutus* and *Rhizopogon roseolus* had no effect on shoot biomass under the given experimental conditions. *S. granulatus* seems to have access to the plant growth limiting resources, whereas the other three fungi do not, or are at least not capable of transferring it to the seedlings. Our findings of species-specific differences are in accordance with the view that mycorrhizal fungi do not always act as mutualists, but rather span a continuum between mutualism and parasitism depending on fungal species genotype, abiotic and biotic environment [35], [36]. In our study, the positive correlation between fungal species number and host productivity was caused by an increasing proportion of samples containing *S. granulatus* in the multi-fungal treatments (Figure 1A). The biomass of seedlings grown with fungal species mixtures did not exceed the biomass of seedlings grown with *S. granulatus* monocultures, indicating a sampling effect rather than niche complementarity. However, we consider this result to be more than an experimental artefact, which a sampling effect often has been claimed to be [37], [38]. The finding that one fungal species improves seedling growth under a given set of environmental variables, whereas others do not, indicates that species composition is more important than species number *per se*. In an ecological context, one could hypothesize that the system is sensitive to one ECM species, and its loss could have substantial functional impact.

We hypothesized that the fungal species’ ability to promote plant growth can be explained by differences in activity of secreted extracellular enzymes, both overall activity and the relative activities of individual enzymes. This was indeed the case for *S. granulatus*, which had the highest overall enzyme activity and induced the largest plant growth (Figures 2A and 4). *Rhizopogon roseolus* and *P. involutus* had a higher overall enzyme activity than *C. geophilum* and non-mycorrhizal root tips, but this did not affect shoot biomass. We therefore conclude that, contrary to our expectations, the eight measured enzyme activities are not suitable to explain differences in host biomass production. Nevertheless, we detected some interesting patterns in enzyme activities (Figure 4). First, ectomycorrhizas did not respond to the drought treatment. We expected that enzymes involved in depolymerizing celluloses and hemicelluloses and thus in mobilizing carbon as glucose would be up-regulated when the C flow from the plant ceases due to drought stress. Such an effect has been shown for field-collected *Lactarius quietus* root tips in a similar situation of C shortage at bud break of oak trees [39] and for *S. granulatus* in a defoliation experiment of *Pinus contorta* [40]. Second, *C. geophilum* had the lowest overall enzyme activity, which did not exceed that of non-mycorrhizal root tips. This is surprising because *C. geophilum* is considered to be a stress resistant species that benefits its plant host even under adverse environmental conditions such as drought stress [41], [42]. Third, we demonstrated that non-mycorrhizal root tips secrete the same set of enzymes as ECM root tips, but that ectomycorrhization profoundly alters relative amounts. Similar patterns have been found for *Populus deltoides* x *P. trichocarpa* hybrids in a recent study by Courty et al. [43]. A major shortcoming of the method of enzyme profiling is that only activities of enzymes on the surface of excised root tips were measured, and there is no information about activities in external mycelium [26]. Nonetheless, in our study, those species that form extensive external mycelium (*S. granulatus*, *P. involutus* and *R. roseolus*) showed higher enzyme activities in excised root tips than *C. geophilum* and non-mycorrhizal root tips.

So far, only two experiments tested the impact of ECM species richness on plant productivity [20], [21]. However, in order to make general conclusions about the role of ECM species richness in regulating plant productivity, more experiments with different hosts and under different environmental conditions are necessary. It has not been investigated yet whether the relationship between ECM species richness and host plant growth can change when climatic conditions change. Our experiment is the first to show that ECM species number does not have an additional benefit to the drought-stressed *P. sylvestris* seedlings compared to the control. The presence of *S. granulatus* improved host shoot biomass particularly under moist conditions (Figure 2A), even though colonization rates did not differ between the two watering regimes (Figure 5). Seedling growth was enhanced by the presence of *S. granulatus* even under the dry soil treatment. This contrasts with the study by Kennedy & Peay [33], who found a positive effect of *Rhizopogon* species on shoot growth of *Pinus muricata* seedlings under moist (13% volumetric water content) but not dry (7% volumetric water content) conditions. The authors concluded that the fungi themselves were affected by water shortage and therefore had limited capacity to provide resources to their host. We recognize that results of such growth chamber experiments are to be interpreted with caution because they can never entirely reproduce natural conditions. For example, in our experiment the pot size might have limited the growth of an extensive mycelium, and therefore constrained the beneficial effect of ECM fungi on water acquisition and uptake. Under natural conditions, some ECM fungi might form mycelial cords - so-called rhizomorphs - that connect numerous plants forming a common mycorrhizal network and allow for water and nutrient exchange [1], [44].

This is one of the first inoculation experiments with potted seedlings where fungal species monocultures are explicitly compared with species mixtures containing up to three species (but see Jonsson et al. [21]). Our study is limited in the number of model species and obviously, it has to be tested to what extent our findings hold for other ECM fungal species. Consecutive experiments may adopt more natural conditions and use a wider array of fungal species, e.g. from different taxonomic groups to check for a phylogenetic signal or from groups of species with different morphological traits such as the presence of an extensive external mycelium and the formation of mycelial cords (exploration types, [45]) to search for causal attributes of different growth responses. Moreover, the assessment of the quantity of external mycelium may also help explain the variable effects of different ECM fungal species. In conclusion, our results suggest that ECM species composition may be important for seedling performance in terms of aboveground biomass.

## Materials and Methods

### Ethics Statement

No specific permits were required for the described study. The soil used was collected from a public forest stand in Valais, Switzerland. The location is not privately owned nor comprises protected area. The fungal strains used in the experiment were from a collection owned by the Swiss Federal Research Institute WSL in Birmensdorf, Switzerland or were provided by INRA UMR1136 Interactions Arbres-Microorganismes in Nancy, France (see below). The experiments did not involve endangered or protected species.

### Study System

Our model system consisted of *Pinus sylvestris* L. seedlings and the following four ECM species: *Cenococcum geophilum* Fr., *Paxillus involutus* (Batsch) Fr., *Rhizopogon roseolus* (Corda) Th. Fr. and *Suillus granulatus* (L.) Roussel. These ECM fungi are widespread species and occur in diverse habitats with *P. sylvestris* as a host, both on seedlings as well as on mature trees [41]. Seedlings were inoculated either with monocultures, or with 2-, 3- or 4-species mixtures (15 combinations in total). An additional treatment included seedlings with no inoculation (non-mycorrhizal treatment). Fourteen weeks after inoculation, two different watering regimes were started with wetting/drying cycles of 3 days. The dry treatment corresponded to 20% of soil water content at field capacity at the time of watering and similarly, the moist treatment to 60%. Each treatment combination was replicated eight times, resulting in a total of 256 pots. Both the soil and the seeds used in this experiment were collected in a mature *P. sylvestris* stand in the Central Alps (Pfynwald, Valais, Switzerland). As inoculum, the following fungal strains were used: *Cenococcum geophilum* Fr. WSL strain 1.58, *Paxillus involutus* (Batsch) Fr. WSL strain 37.6, *Rhizopogon roseolus* (Corda) Th. Fr. WSL strain 97.03 and *Suillus granulatus* (L.) Roussel. strain FROI S101 provided by INRA UMR1136 Interactions Arbres-Microorganismes. The *C. geophilum*, the *R. roseolus* and the *S. granulatus* strain were isolated from sclerotia and sporocarps, respectively, sampled in the same *P. sylvestris* stand where the soil and the seeds for this experiment originated from. The *P. involutus* strain was isolated from a sporocarp sampled in a coniferous forest in Sâles, Fribourg, Switzerland. Voucher material is deposited in the mycology collection of the Swiss Federal Research Institute WSL in Birmensdorf, Switzerland.

### Inoculation

Mycelium plugs of each fungus were grown in liquid cultures with Marx-Melin-Norkrans medium [46]. For *C. geophilum*, a slightly different growing medium with casein instead of malt extract was used [47]. To propagate the inoculum, the cultures were mixed with a blender every 3–4 weeks and transferred into new Erlenmeyer flasks containing sterile growing medium. Seeds of *P. sylvestris* were surface sterilized with 35% H_2_O_2_ for 30 minutes and sown in 300 ml pots containing a 1∶2 mixture of autoclaved (120 °C, 20 min) quartz sand and sieved topsoil of a mature *P. sylvestris* stand. The autoclaved soil/sand mixture had a pH_CaCl_2_ of 4.4, a C∶N ratio of 15.7 and available P content of 7.1 mg/kg dry soil. The pots were placed in a growth chamber with day/night cycles of 15 h/9 h, temperature of 26 °C/20 °C, respectively, relative humidity of 60%, and mean irradiance of 750 lux during the day cycles. These growth chamber settings were chosen to provide optimal conditions for the growth of the ECM fungi. Pots were watered with de-ionized water every third day._

After twelve weeks, seedlings from 240 pots were inoculated as follows: Excess medium was decanted from the Erlenmeyer flasks and the mycelium was fragmented and homogenized with a blender for 2–3 seconds. Then the mixtures of each of the 15 species combinations containing equal volumes of the respective species slurry were prepared. Seedlings were carefully excavated from soil, and roots of two selected individuals were sprinkled with the amount of 3 ml from one of the 15 inoculum mixtures by means of a pipette. Subsequently, the two seedlings were re-potted into the same substrate and pot. The decanted excess medium was autoclaved and 3 ml of it was applied to two seedlings of each of the remaining 16 pots as a negative control (non-mycorrhizal treatment). All pots were filled with sterile substrate to achieve the same weight. In order to prevent desiccation, pots were covered with a thin layer of sterilized perlite. The whole procedure was carried out in a laminar flow hood to avoid contamination. During the first two weeks after inoculation, the relative humidity in the growth chamber was raised to 80% to prevent drought stress in re-potted seedlings. Watering of the pots with de-ionized water was continued every third day. Every four weeks, the pots were rearranged in a systematic manner. Fourteen weeks after inoculation, two different watering treatments were started. Soil water content at field capacity was determined gravimetrically for six spare pots. Pots were then watered to a weight that corresponded to the sum of dry weight of the soil in a pot, weight of the pot and perlite, and 20% and 60% of the weight of soil water content at field capacity, for the dry and moist treatment respectively. Subsequently, pots were allowed to dry out for 3 days and then watered again, and so forth. A previous experiment showed that watering to approximately 20% of the weight of soil water content at field capacity was necessary to prevent seedlings to reach the wilting point within 3 days. Since seedling weight accounted only for a small proportion of total pot weight, differences in pot weight due to variable growth of different species treatments were not taken into account.

### Harvesting, Assessment of Successful Infection and Enzyme Activity Measurements

27–30 weeks after inoculation, the seedlings were harvested and roots were carefully washed under tap water. Roots of two seedlings per pot were cut into 3–5 cm pieces, and tips of a randomly selected subset of root pieces were inspected for mycorrhization until at least 300 tips were counted. Pooled roots were checked for the presence/absence of the initially inoculated species based on their morphological characteristics. Almost no dead or senescent root tips were found, ruling out the possibility that one of the species has infected the roots but has disappeared until the time of harvest. None of the control pots (non-mycorrhizal treatment) were colonized by contaminant ECM fungi. Shoots and roots were dried at 70 °C for 48 h. Dry weight was determined and divided by two in order to get a mean value per seedling and pot for use in further data analysis. During the course of the experiment, seedlings in three pots of the dry treatment died, one containing *R. roseolus*, another *R. roseolus* and *S. granulatus*, and a third with the 4-species mixture. At harvest, only three out of fifteen 4-species mixture pots contained all four species that had initially been inoculated. Therefore, the 4-species mixture treatment was omitted from further analysis. Each species had been inoculated in 128 pots, but at harvest, only 124, 66, 118 and 119 pots contained *C. geophilum*, *P. involutus*, *R. roseolus* and *S. granulatus*, respectively.

For the measurement of the activity of eight key extracellular enzymes, seven root tips from each fungal monoculture pot were randomly selected immediately after washing the roots at the time of harvest. They were placed on a 96-well microtitration plate containing enzyme substrates and incubation buffers. Seven fluorescence tests were performed successively on the same microtitration plate, utilizing enzyme substrates based on methylumbelliferone (MU) and 7-amino-4-methylcoumarine (AMC): MU-phosphate (MU-P) for the detection of acid phosphatase (EC 3.1.3.2), MU-β-D-glucopyranoside (MU-G) for β-glucosidase (EC 3.2.1.3), MU-*N*-acetyl-β-D-glucosaminide (MU-NAG) for chitinases (EC 3.2.1.52), MU-β-D-glucuronide hydrate (MU-GU) for glucuronidase (EC 3.2.1.31), MU-β-D-xylopyranoside (MU-X) for xylosidase (EC 3.2.1.37), MU-β-D-cellobioside (MU-C) for cellobiohydrolase (EC 3.2.1.91) and L-leucine-AMC (Leu-AMC) for leucine aminopeptidase (EC 3.4.11.1) activities. A photometric test with 2,2’-azinobis-3-ethylbenzothiazoline-6-sulfonate (ABTS) solution was performed to detect laccase (EC 1.10.3.2) activity. Depending on substrate, incubation times were 20 min (MU-P), 15 min (MU-G), 15 min (MU-NAG), 30 min (MU-GU), 50 min (MU-X), 30 min (MU-C), 60 min (Leu-AMC) and 60 min (ABTS). Details concerning stock solutions of substrates, calibration solutions and incubation and stopping buffers are described in detail in [25]. Measurements were carried out with a Tecan Infinite M200 microplate reader (Tecan GmbH, Germany) with an excitation wavelength of 368 nm and an emission wavelength of 465 nm for the fluorescence tests, and an absorbance measurement wavelength of 425 nm for the photometric test. The projected area of the mycorrhizal tips was measured by scanning the 96-well plate followed by image analysis using WinRhizo Software (Regent Instruments Inc., Canada). Measured activities were standardized by the projected area of the root tips and the incubation time. For more than half of the root tips, especially those with *R. roseolus* and non-mycorrhizal tips, laccase activity was below detection level, and therefore, this enzyme was omitted from further analysis.

### Data Analysis

Effects of presence and identity of ECM fungi and watering regime on shoot biomass were investigated using two separate generalized linear models (GLMs) with normal distribution of errors and identity link (which is equivalent to normal regression, and resulted in slightly lower AIC values than the log link). To improve the model fit, shoot biomass was log-transformed. For the analysis of root/shoot ratio, a GLM with normal distribution and log link was used. Overall enzyme activities were analyzed by a 2-way analysis of variance and *a posteriori* differences between factor levels were assessed by Tukey’s HSD test. To check for overyielding, shoot biomass of seedlings grown in monocultures of *S. granulatus* - the fungal species with the most growth-promoting effect - was compared to that of seedlings in species mixtures using pairwise, two-sided *t*-tests with Holm’s correction for multiple comparisons. Colonization rate between the moist and the dry treatment was compared with a two-sided Welch *t*-test. To visualize the relationship between colonization rate and shoot biomass for different treatment groups, lines based on locally weighted scatterplot smoothing (LOWESS, see [48]) were drawn in the scatterplot. The statistical computing system R version 2.11.0 was used for data analyses [49].
